# Successful Treatment of Descending Necrotizing Mediastinitis by Thoracotomy Using a Pedicled Omental Flap: A Case Report

**DOI:** 10.7759/cureus.75211

**Published:** 2024-12-06

**Authors:** Yasuaki Tomioka, Kenta Manabe, Tomohiro Hayashida, Eiji Yamada, Masahiko Muro

**Affiliations:** 1 Thoracic Surgery, Fukuyama City Hospital, Fukuyama, JPN; 2 Thoracic Surgery, Okayama University Hospital, Okayama, JPN; 3 Cardiovascular Surgery, Fukuyama City Hospital, Fukuyama, JPN

**Keywords:** descending necrotizing mediastinitis, empyema, mediastinal infection, omental flap, perivascular involvement, staged surgery

## Abstract

Descending necrotizing mediastinitis (DNM) is a severe, life-threatening infection that requires prompt diagnosis and aggressive surgical intervention. Management is particularly challenging when the condition is complicated by bilateral empyema and perivascular involvement. A 73-year-old woman presented with septic shock several days after experiencing pharyngeal pain. Initial computed tomography revealed a deep neck infection extending into the mediastinum, with bilateral empyema. Despite emergency thoracoscopic drainage, follow-up imaging revealed the progression of the infection along the perivascular spaces. A staged surgical approach culminating in thoracotomy with extensive debridement and pedicled omental flap coverage from the dorsal pulmonary hilum to the periaortic area was used. Although postoperative management was prolonged and required tracheostomy and extended vasopressor support, the patient eventually recovered and was discharged for rehabilitation. This case demonstrates that while initial thoracoscopic drainage may be appropriate for critically ill patients with DNM, the progression of infection along the perivascular spaces may necessitate escalation to open surgical debridement with omental flap coverage. Careful monitoring of disease progression and appropriate modifications of surgical strategies are crucial for successful treatment.

## Introduction

Descending necrotizing mediastinitis (DNM) is a rare but severe infection that typically originates from the oropharyngeal or odontogenic sources and rapidly spreads to the mediastinum [1,2]. Early diagnosis using computed tomography (CT) and prompt surgical intervention are crucial, as DNM can rapidly progress to septic shock and organ failure [1]. Despite advances in treatment, DNM remains a serious condition, with mortality rates ranging from 5.6% to 28.5% [1,3].

Management of DNM typically requires a multidisciplinary approach that combines aggressive surgical debridement with appropriate antibiotic therapy [4]. The most common causative organisms are *Streptococcus* species and anaerobic bacteria, reflecting the oral cavity microflora [1]. Initial empiric antibiotic therapy typically includes broad-spectrum coverage, with carbapenems and clindamycin being frequently used [1]. Various surgical approaches have been described, including transcervical, thoracoscopic, and combined procedures, depending on the extent of mediastinal involvement [3,5]. Biomarkers, particularly C-reactive protein (CRP), have been reported as important prognostic indicators, with markedly elevated levels associated with poor outcomes [6,7]. In cases of extensive infection with bilateral empyema, some studies have reported successful outcomes using pedicled omental flap transposition, which can help control sepsis and provide additional vascularization to the infected area [8,9].

Herein, we present a case of severe DNM complicated by bilateral empyema that was successfully managed with a combination of thoracoscopic drainage, open thoracotomy, and omental flap transposition, highlighting the importance of aggressive surgical intervention and comprehensive postoperative care to achieve favorable outcomes.

## Case presentation

A 73-year-old woman was admitted to our hospital with progressive deterioration after several days of pharyngeal pain. On initial presentation at the previous hospital, she exhibited signs of septic shock with systolic blood pressure in the 70s mmHg and severe respiratory distress, requiring high-flow oxygen supplementation. Despite fluid resuscitation, the patient’s hemodynamic status remained unstable, necessitating catecholamine support. Initial CT at another hospital revealed low-density areas with air tracking from the right cervical region extending into the mediastinum, suggesting deep neck infection with mediastinitis as the cause of septic shock.

**Figure 1:**
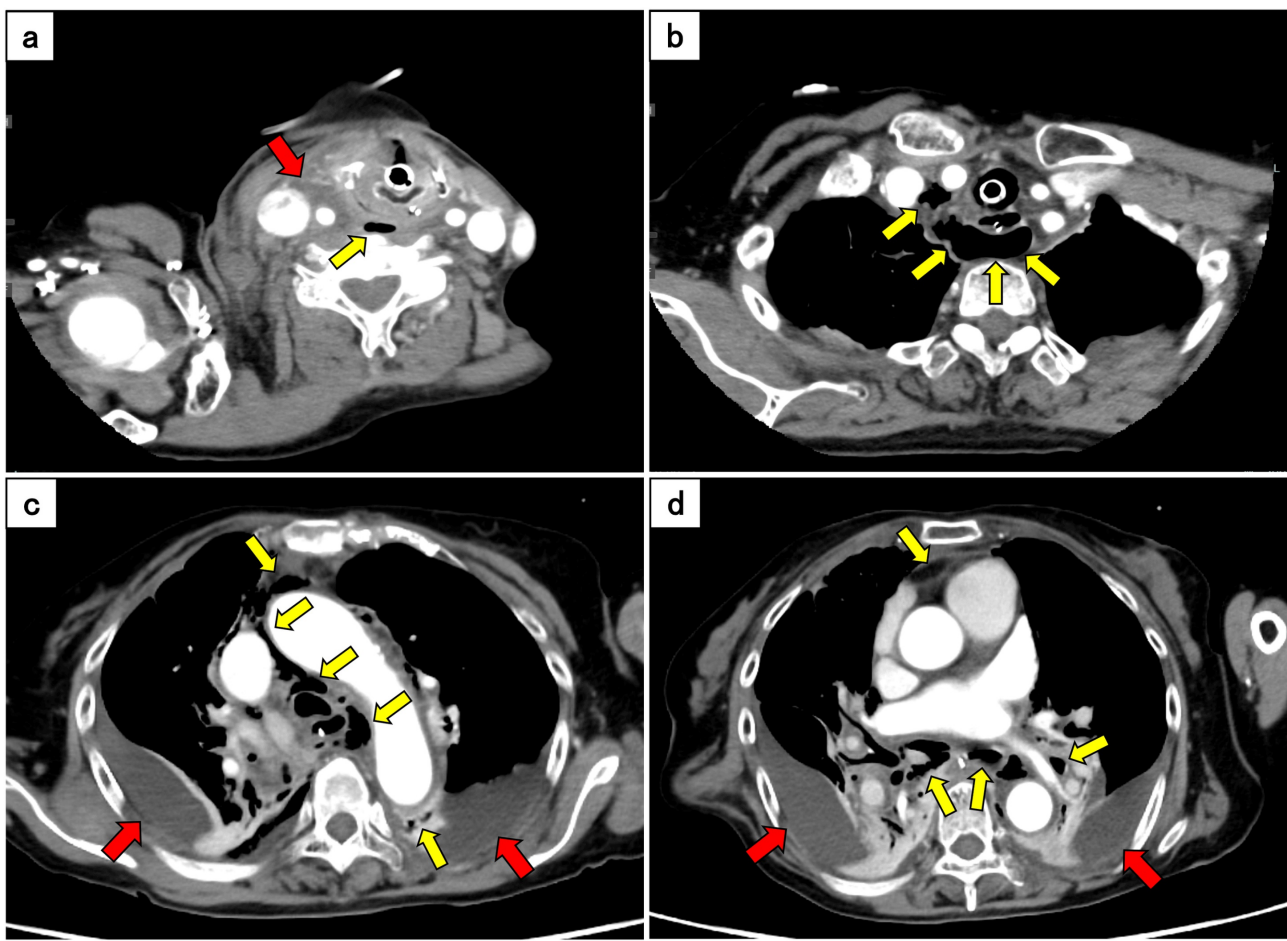
Contrast-enhanced computed tomography on admission in the axial plane (a) Low-density areas in the carotid and retropharyngeal spaces (red arrow) with air tracking extending from the retropharyngeal space to the mediastinum. (b-d) Extensive pneumomediastinum (yellow arrows) with bilateral encapsulated pleural effusion (red arrows).

Upon arrival at our hospital, the patient's condition deteriorated with respiratory acidosis and hypoxemia requiring immediate endotracheal intubation and mechanical ventilation. The patient was empirically treated with meropenem. Hemodynamic instability persisted despite the initiation of noradrenaline and vasopressin, necessitating additional epinephrine infusion and albumin administration to maintain systolic blood pressure above 90 mmHg. Contrast-enhanced CT revealed low-density areas with air tracking from the carotid and retropharyngeal spaces to the mediastinum accompanied by bilateral encapsulated pleural effusions (Figure 1). Based on these findings, the patient was diagnosed with DNM complicated by bilateral empyema, and emergency surgery was planned.

**Figure 2:**
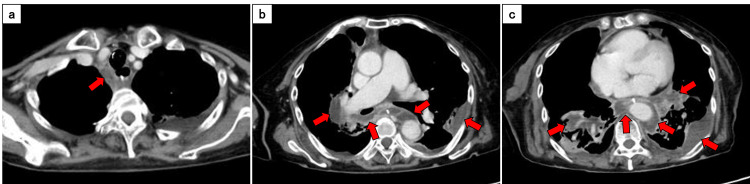
Contrast-enhanced computed tomography on postoperative day 8 in the axial plane (a-c) Slight progression of low-density areas (red arrows) suggestive of abscess formation around the mediastinum, hilar region, thoracic aorta, and right hypopharyngeal region. Note the mild increase in the left pleural effusion.

The patient's unstable condition precluded single-lung ventilation with a double-lumen tube; therefore, we performed video-assisted thoracoscopic surgery (VATS) including right mediastinotomy and empyema debridement, along with right cervical drainage under general anesthesia using a bronchial blocker. Left thoracic drainage was performed concurrently. Pleural fluid culture revealed typical oral flora, including *Streptococcus constellatus*/*milleri* (3+), *Prevotella* species (3+), and *Peptostreptococcus asaccharolyticus* (2+), confirming the oropharyngeal origin of the infection. On postoperative day 8, due to progressive empyema, we performed additional VATS for right-sided empyema debridement and placed a second thoracic drainage catheter on the left side (Figure 2).

**Figure 3:**
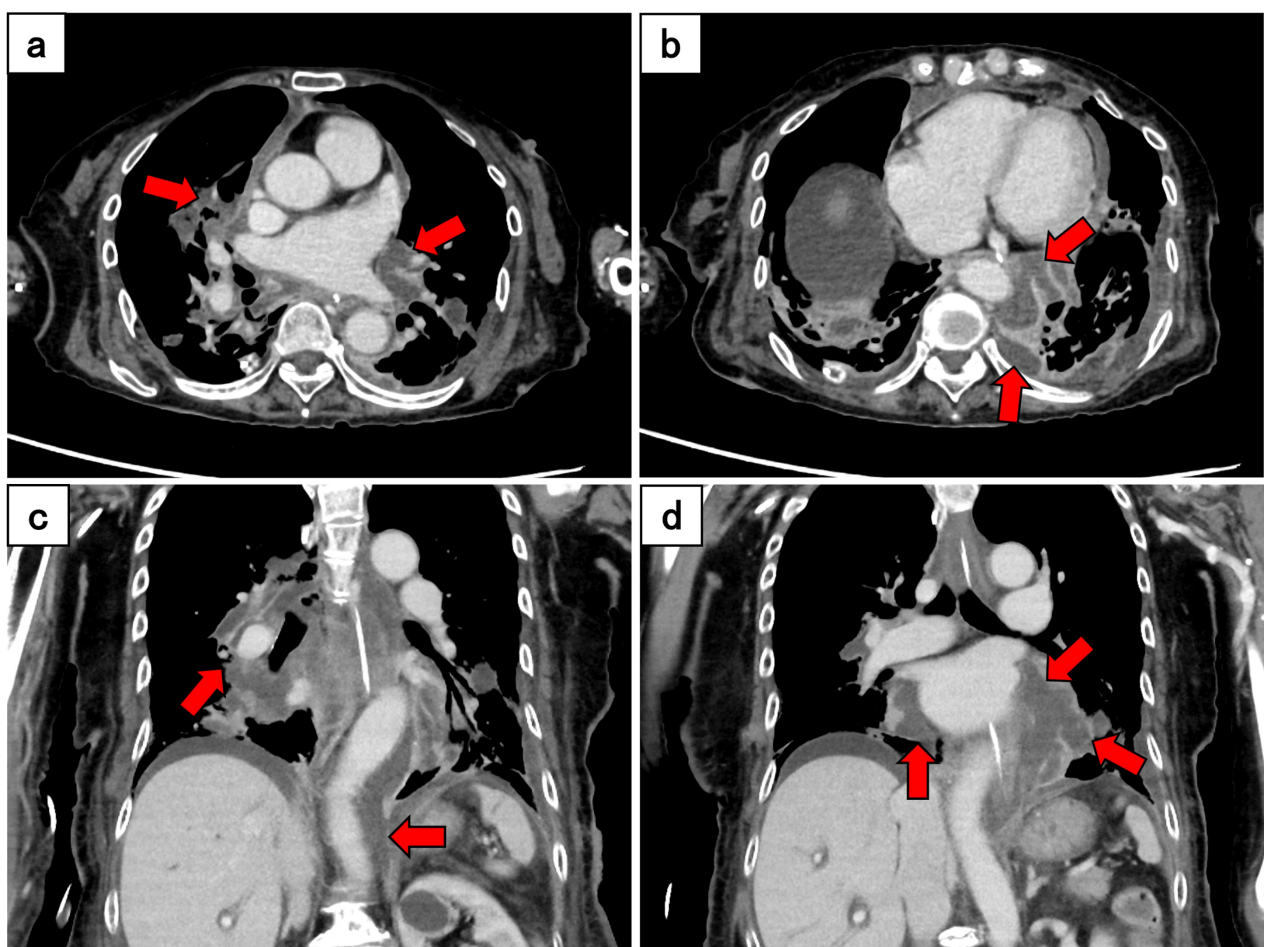
Contrast-enhanced computed tomography on postoperative day 16 in the axial (a and b) and coronal (c and d) planes Expanding low-density areas (red arrows) around the aorta and bilateral hilar vessels indicate abscess progression. On the right side, low-density areas extend to the superior mediastinum and interlobar fissures (upper-lower and middle-lower). The infection involves the vascular sheaths of the pulmonary arteries and veins. On the left side, low-density areas were present around the inferior pulmonary vein and the lung base. Aortic involvement extended to the diaphragmatic crura (red arrows). A small amount of ascitic fluid was also observed.

The patient remained catecholamine-dependent despite a slight improvement in inflammatory markers after the second operation. The antibiotic regimen was changed to ampicillin/sulbactam on postoperative day 19 based on clinical response and culture results. Follow-up contrast-enhanced CT revealed expanding low-density areas around the aorta and bilateral hilar vessels, suggesting abscess progression. The infection extended along the pulmonary arterial and venous sheaths, with involvement of the superior mediastinum, interlobar fissures on the right side, and the inferior pulmonary vein and lung base on the left side. The aortic involvement extended to the level of the diaphragmatic crura (Figure 3).

**Figure 4:**
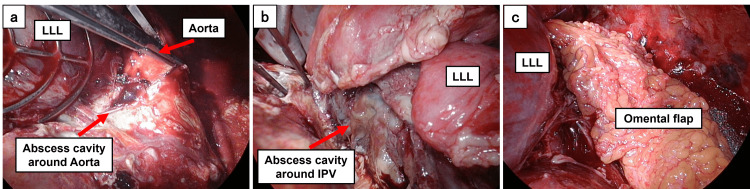
Intraoperative findings (a) Incision and drainage of the periaortic abscess cavity. (b) Incision and drainage of the peri-IPV abscess cavity. (c) Coverage with a pedicled omental flap from the dorsal pulmonary hilum to the periaortic area. IPV: inferior pulmonary vein; LLL: left lower lobe

On postoperative day 16 (after the initial surgery), we performed a left thoracotomy for mediastinal and perivascular drainage, coverage using a pedicled omental flap from the dorsal pulmonary hilum to the periaortic area, and thoracoscopic right empyema debridement. After extensive mediastinal and left thoracic dissections, a median laparotomy was performed to create a pedicled omental flap. Since the infection extended along the aorta to the level of the aortic hiatus, we enlarged the hiatus to an appropriate size to prevent omental strangulation and passed the omental flap through it. The flap was then used to cover the affected areas from the dorsal pulmonary hilum through the periaortic region to the level of the aortic hiatus (Figure 4).

**Figure 5:**
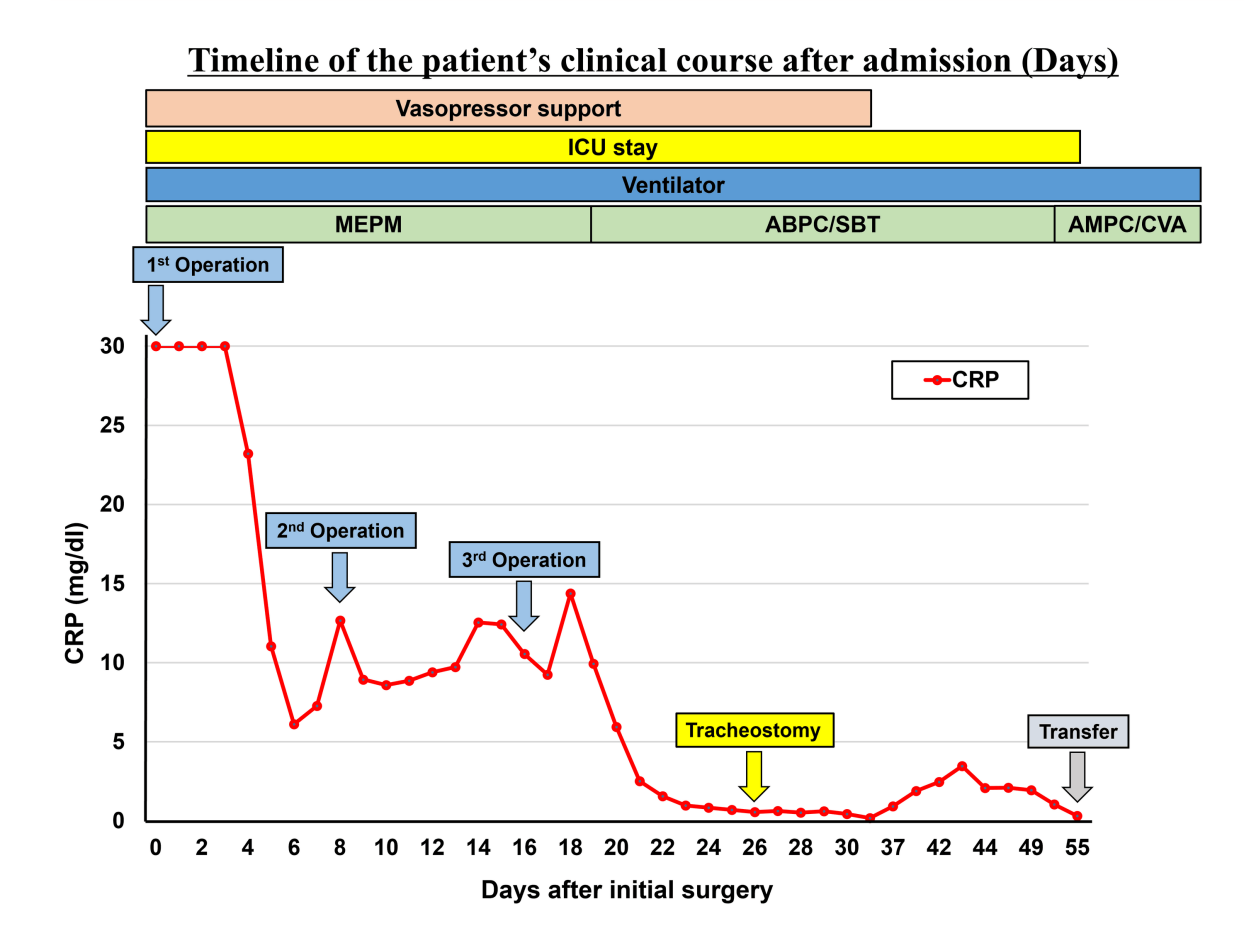
Timeline of the patient’s clinical course ABPC/SBT: ampicillin/sulbactam; AMPC/CVA: amoxicillin/clavulanic acid; MEPM: meropenem

Postoperatively, the patient continued to receive antibiotics, and respiratory and circulatory management with ventilators and vasopressors. Multiple attempts at ventilator weaning were unsuccessful due to respiratory distress and tachypnea, necessitating tracheostomy on postoperative day 26. The vasopressor support was discontinued on postoperative day 33. The patient was transferred to a rehabilitation facility on postoperative day 55 (Figure 5).

## Discussion

This case highlights two important aspects in the management of severe DNM complicated by bilateral empyema. First, prompt diagnosis and aggressive surgical intervention are crucial, even in patients with severe septic shock who require multiple vasopressors [1,10]. Second, careful monitoring of disease progression and readiness to escalate surgical intervention are essential, particularly when perivascular spread is identified.

In the present case, a staged surgical approach was initially selected because of the patient's critical condition. Despite markedly elevated CRP levels (>30 mg/dL) at presentation, which have been associated with a poor prognosis [6,7], initial thoracoscopic drainage provides temporary source control [10]. However, follow-up imaging revealed the progression of the infection along the perivascular spaces (Figures 2-3). This progression pattern demonstrates that, when the infection extends along the perivascular spaces, thoracoscopic drainage alone may be insufficient, necessitating extensive open surgical debridement [3]. The use of a bronchial blocker may be effective when double-lumen tube placement is contraindicated due to the patient's unstable condition.

The decision to perform omental flap transposition was based on the persistent infection and extensive perivascular involvement (Figure 3). The rich vascularity and immunological properties of the omentum make it suitable for managing infected spaces [8], and its excellent coverage and mobility make it ideal for mediastinal reconstruction [11]. Strategic placement from the dorsal pulmonary hilum to the periaortic area provided effective coverage of the infected spaces (Figure 4), supporting previous reports of successful omental flap transposition in complex DNM cases [8,12].

The postoperative management was characterized by prolonged respiratory failure requiring tracheostomy and extended vasopressor dependence (Figure 5). These intensive care requirements are consistent with previous reports on severe DNM [7,13]. Our experience demonstrates that appropriate airway management strategies and intensive care support are essential for the successful treatment of severe DNM.

## Conclusions

This case illustrates that while initial thoracoscopic drainage may be appropriate for critically ill patients with DNM, the progression of infection along the perivascular spaces may necessitate escalation to open surgical debridement with omental flap coverage. Careful monitoring of disease progression using CT and appropriate modification of surgical strategies are essential for successful treatment.
